# Antioxidants Abrogate Alpha-Tocopherylquinone-Mediated Down-Regulation of the Androgen Receptor in Androgen-Responsive Prostate Cancer Cells

**DOI:** 10.1371/journal.pone.0151525

**Published:** 2016-03-17

**Authors:** Alexandra M. Fajardo, Debra A. MacKenzie, Sarah L. Olguin, John K. Scariano, Ian Rabinowitz, Todd A. Thompson

**Affiliations:** 1 Department of Pharmaceutical Sciences, University of New Mexico College of Pharmacy, Albuquerque, New Mexico, United States of America; 2 Department of Pathology, University of New Mexico School of Medicine, Albuquerque, New Mexico, United States of America; 3 Division of Hematology/Oncology, Department of Internal Medicine, University of New Mexico School of Medicine, Albuquerque, New Mexico, United States of America; 4 University of New Mexico Comprehensive Cancer Center, University of New Mexico, Albuquerque, New Mexico, United States of America; Thomas Jefferson University, UNITED STATES

## Abstract

Tocopherylquinone (TQ), the oxidation product of alpha-tocopherol (AT), is a bioactive molecule with distinct properties from AT. In this study, AT and TQ are investigated for their comparative effects on growth and androgenic activity in prostate cancer cells. TQ potently inhibited the growth of androgen-responsive prostate cancer cell lines (e.g., LAPC4 and LNCaP cells), whereas the growth of androgen-independent prostate cancer cells (e.g., DU145 cells) was not affected by TQ. Due to the growth inhibitory effects induced by TQ on androgen-responsive cells, the anti-androgenic properties of TQ were examined. TQ inhibited the androgen-induced activation of an androgen-responsive reporter and inhibited the release of prostate specific antigen from LNCaP cells. TQ pretreatment was also found to inhibit AR activation as measured using the Multifunctional Androgen Receptor Screening assay. Furthermore, TQ decreased androgen-responsive gene expression, including TM4SF1, KLK2, and PSA over 5-fold, whereas AT did not affect the expression of androgen-responsive genes. Of importance, the antiandrogenic effects of TQ on prostate cancer cells were found to result from androgen receptor protein down-regulation produced by TQ that was not observed with AT treatment. Moreover, none of the androgenic endpoints assessed were affected by AT. The down-regulation of androgen receptor protein by TQ was abrogated by co-treatment with antioxidants. Overall, the biological actions of TQ were found to be distinct from AT, where TQ was found to be a potent inhibitor of cell growth and androgenic activity in androgen-responsive prostate cancer cells.

## Introduction

**Fig 1:**
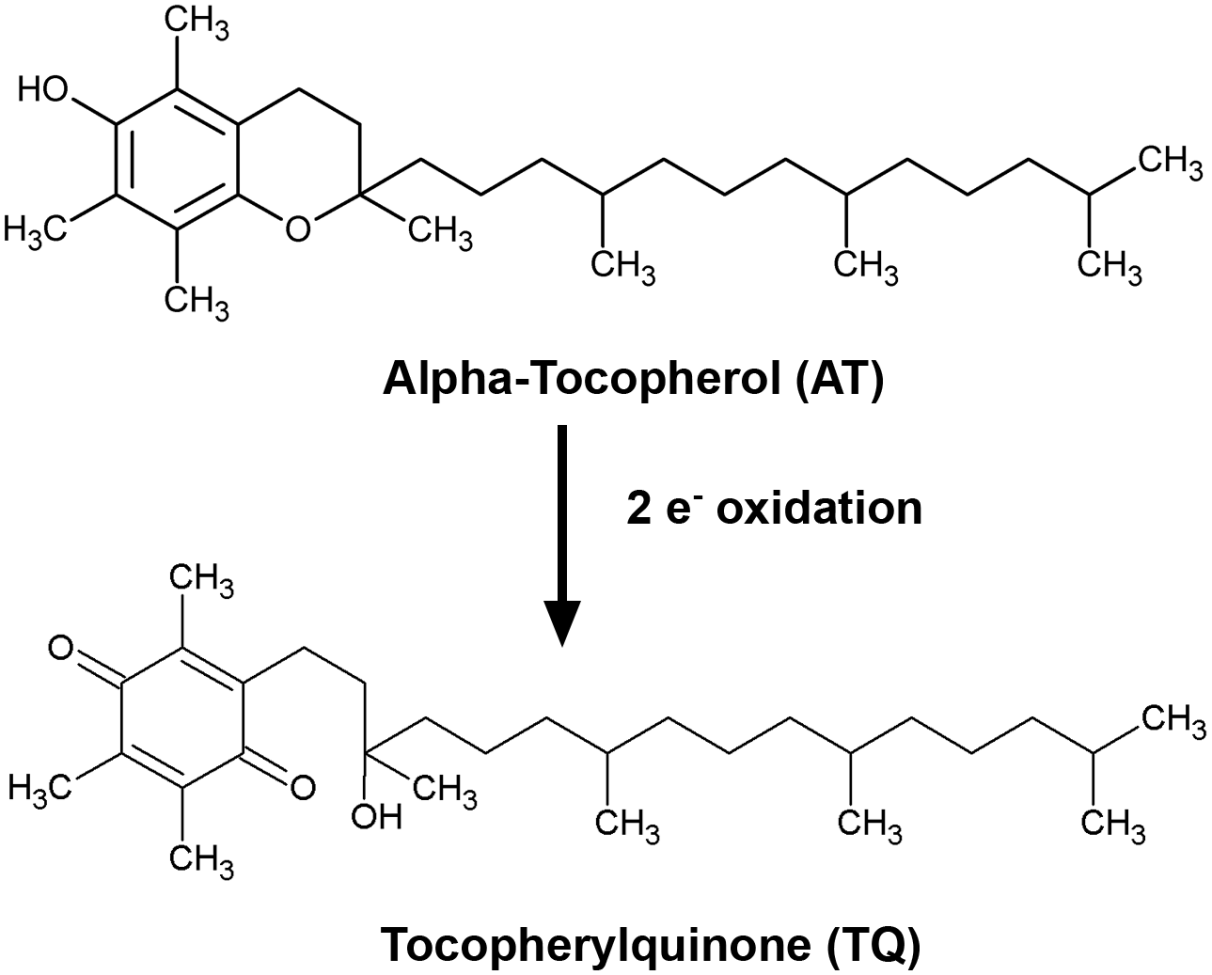
Alpha-tocopherylquinone (TQ) is produced by the two-electron oxidation of the chromanol moiety of alpha-tocopherol (AT).

The role of antioxidant action in cancer development and progression is unclear. Vitamin E is a family of naturally occurring dietary factors (e.g., α-,β-,γ-,δ-tocopherols and -tocotrienols) with a major biologically active form recognized as RRR-α-tocopherol (RRR-AT) [1,2]. α-tocopherol (AT) acts primarily as an antioxidant, reducing cellular oxidative damage produced by oxidized lipids [1,2]. The major oxidation product of AT is α-tocopherylquinone (TQ), which is formed by the two-electron oxidation of the chromanol moiety of AT (Fig 1). TQ has distinct chemical properties compared to AT with a unique spectrum of biomolecular actions [3]. For example, TQ has been shown to inhibit the growth of colon cancer cells, whereas AT was not observed to alter the growth of these cells [4]. Although TQ is established as a bioactive quinone for some cancers, its action on prostate cancer cell growth and androgenic pathways in prostate cancer cells are unknown.

The AR is a necessary contributor to prostate cancer development and is recognized as a meaningful target for prostate cancer prevention and treatment [5]. This is supported by the importance of the AR in prostate cancer progression [6–8] and from the outcome of studies using inhibitors of testosterone metabolism to prevent prostate cancer development [8,9]. The AR is a member of the steroid hormone/nuclear receptor superfamily [10], which acts as a ligand-activated transcription factor for genes involved in the growth, survival, and differentiation of the prostate [11]. In addition, AR activity contributes to the development, progression, and maintenance of prostate cancer [7,12]. Clinically, down-regulation of AR activation is achieved through a number of means that include the direct interference of androgen binding to the AR as with AR antagonists, by decreasing dihydrotestosterone production with 5-alpha-reductase inhibitors, or by decreasing the production of testosterone by gonadotropin-releasing hormone agonists and CYP17A1 inhibitors [7,12,13]. These strategies do not directly target the expression of AR protein and thus, under these interventions, the AR remains functional.

Information on the biological actions of TQ are limited compared to the more extensive investigations on AT. To date, there are no studies examining the effect of TQ on cell growth and androgenic activity in prostate cancer cells. However, modulation of AR activity by AT-related agents has been reported. The mechanism of androgenic inhibition by these chemicals may be direct or indirect. For example, we have previously shown that the chromanol moiety of AT blocks androgenic activity by competitive inhibition of androgen binding to the AR [14]. Direct inhibition of the AR has been observed with AT succinate, a more water soluble ester derivative of AT, which has been shown to down-regulate AR protein in androgen-responsive prostate cancer cells [15]. Because AT analogs and derivatives have shown biological action against prostate cancer cells, whereas the actions of the oxidation product of AT in prostate cancer are unknown, we aimed to determine if TQ possessed unique biologic action in prostate cancer cells. In this study, the effects of both AT and TQ on prostate cancer cell proliferation, anti-androgenic activity, and potential mechanism of AR protein down-regulation were evaluated. Compared to AT, TQ was found to have distinctive properties on androgen-responsive prostate cancer cell lines with notable actions on the expression of the AR. Antioxidants were found to modify the effects of TQ on these cells. This study begins to elucidate the mechanism of TQ on inhibiting AR protein expression that may be through its activity to alter the redox state in prostate cancer cells.

## Results

### Inhibition of cell proliferation in androgen-sensitive prostate cancer cell lines by TQ

**Fig 2:**
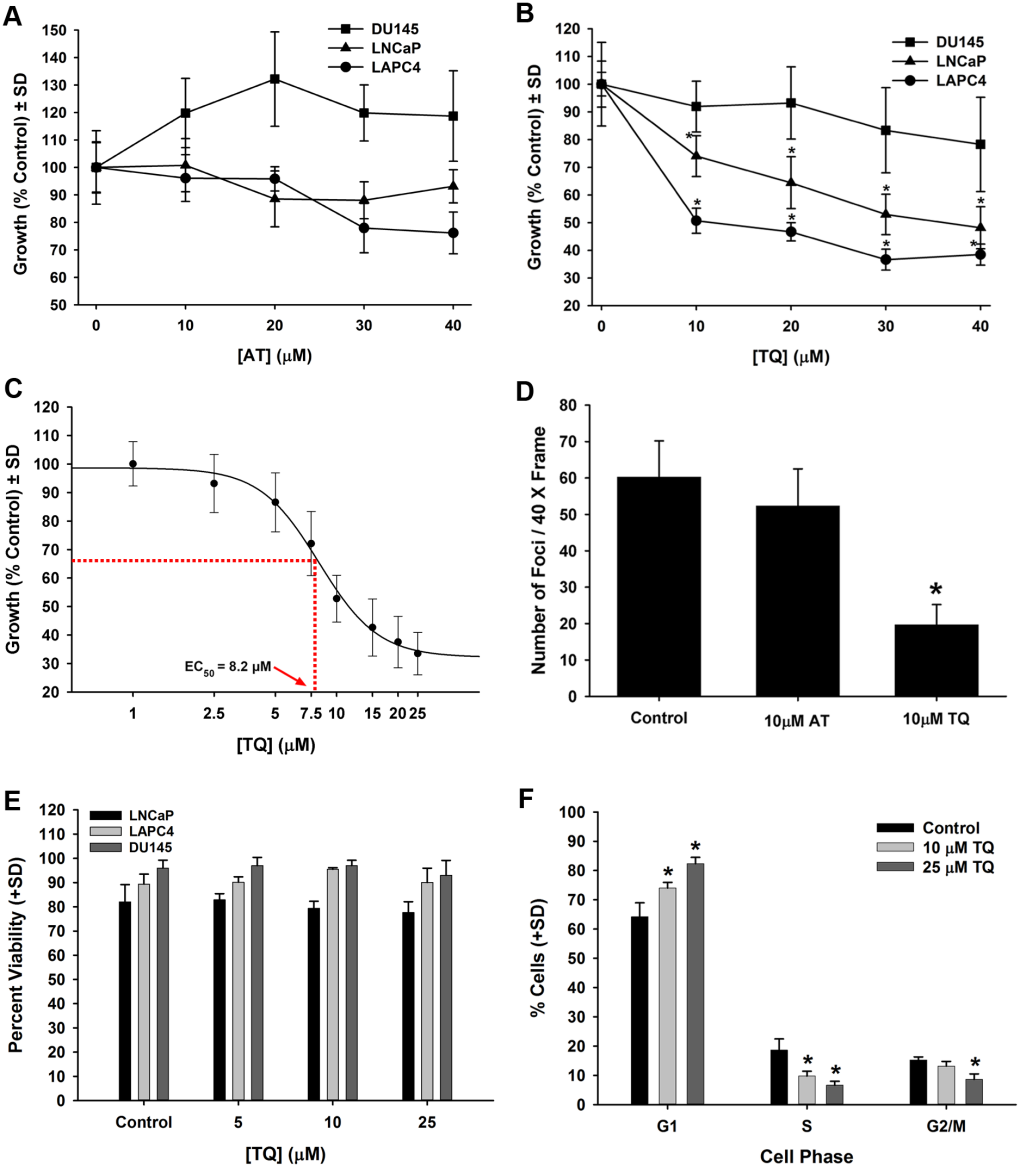
**A-F**. TQ-mediated inhibition of cell proliferation, but not viability, in prostate cancer cell lines. (A) AT growth analysis in androgen-sensitive LAPC4 and LNCaP cells and androgen-independent DU145 prostate cancer cells. (B) Dose-response of DU145, LAPC4, and LNCaP cell growth in cells treated with TQ for 4 d. (C) TQ EC_50_ determination for LAPC4 cell growth. (D) Determination of LNCaP growth foci following treatment with either 10 μM AT or 10 μM TQ for 5 d. (E) Viability analysis in DU145, LAPC4 and LNCaP cells at 5, 10, and 25 μM TQ for 48 h with no decrease in cell viability. (F) Cell cycle analysis (i.e., DNA content) in LNCaP cells treated with 10 and 25 μM TQ showing a G1 arrest. * *P*<0.05.

Previous studies have demonstrated that ester-conjugated, water soluble VE analogs (e.g., vitamin E succinate) can inhibit prostate cancer cell growth in culture [15,16]. Because of concerns regarding the production of the physiologically active free forms of AT and TQ from the conjugated forms, the unconjugated, free forms of these agents were investigated. As the free forms of AT and TQ have low aqueous solubility, a carrier-based delivery method was developed to administer the free forms of these agents in cell culture, as described in the *Materials and Methods*. AT treatment had minimal effects on the growth of androgen-sensitive LAPC4 and LNCaP cells as well as androgen-independent DU145 prostate cancer cells after treatment with concentrations of AT up to 40 μM (Fig 2A). In contrast, TQ treatment produced a dose-dependent growth decrease of androgen-responsive LAPC4 and LNCaP prostate cancer cells, whereas the growth of DU145 cells was not affected by TQ at doses up to 40 μM (Fig 2B). In a more resolved dose-response with LAPC4 cells, the EC_50_ of TQ for inhibiting cell growth was found to be 8.2 μM (Fig 2C). Similarly, in a focus forming analysis of LNCaP cell growth, TQ was found to inhibit focus formation, whereas AT did not affect focus formation (Fig 2D). To determine if the inhibition of cell growth by TQ was in part due to effects on cell viability, DU145, LAPC4 and LNCaP cell viability was measured by hemocytometry and trypan blue exclusion. The viability of cells treated with 5, 10, and 25 μM TQ did not differ from vehicle-treated cells (Fig 2E). In addition, a flow cytometric-based propidium iodide exclusion analysis of LNCaP cells showed 92.4% (±1.4%) viability in vehicle-treated controls and 93.2% (±1.2%) for 25 μM TQ-treated LNCaP cells. For DU145 cells, 94.7% (±1.0%) viability was observed in vehicle-treated controls and 94.4% (±1.3%) for 25 μM TQ-treated cells by propidium iodide exclusion, further supporting that TQ does not acutely affect cellular viability at doses up to 25 μM. LNCaP cells treated with either 10 or 25 μM TQ showed an increased number of cells in G1 phase with a concurrent decrease of S phase cells (Fig 2F). This data supports that TQ inhibits androgen-sensitive prostate cancer cell proliferation through a G1 arrest at doses that do not decrease the viability of these cells.

### TQ, not AT, treatment decreases AR activity and AR regulated mRNA transcript levels

**Fig 3:**
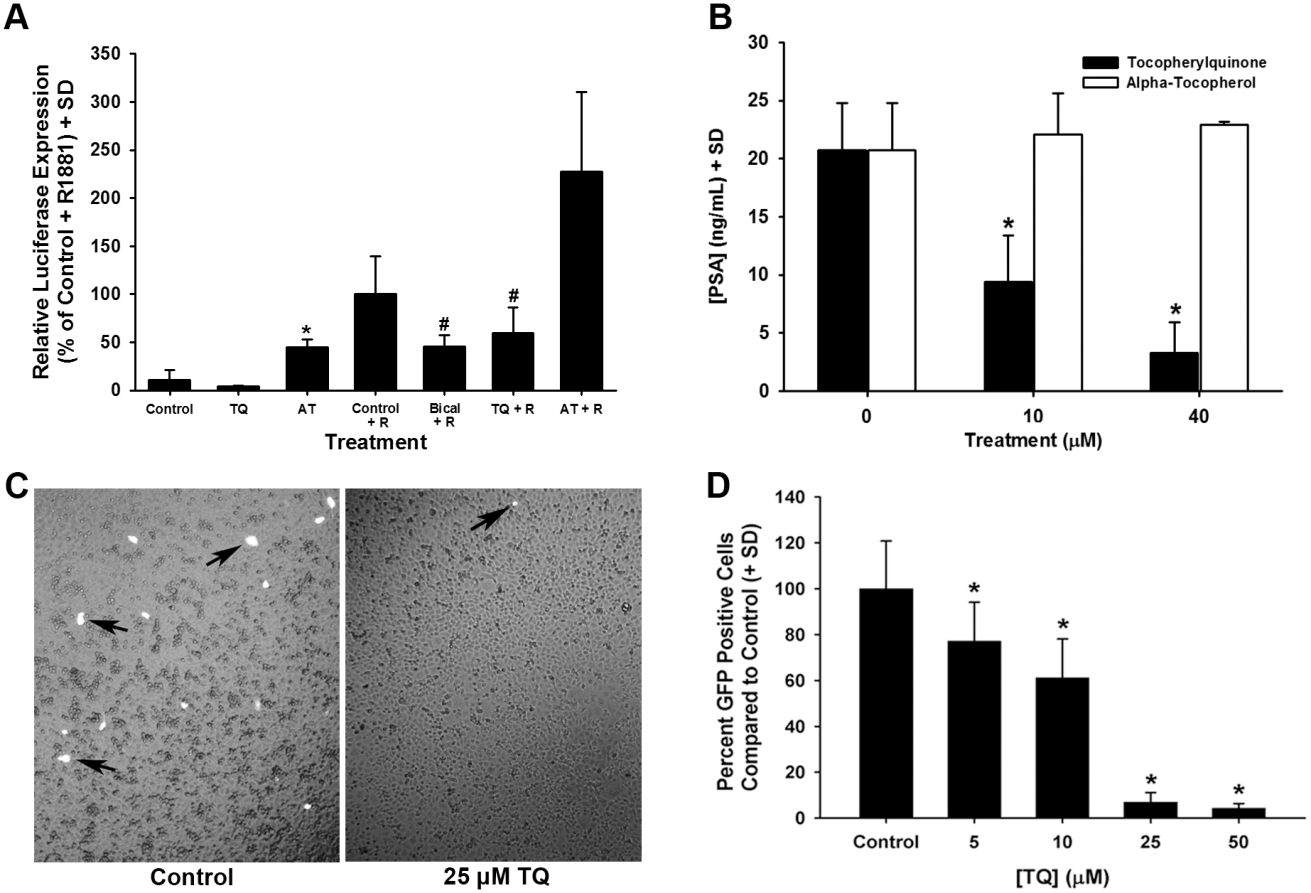
**A-D**. Inhibition of androgenic responses in LNCaP cells by TQ treatment. (A) Androgen-induced [i.e., R1881 (R)] luciferase expression from an androgen-sensitive promoter measured after TQ or AT treatment for 48 h. Bicalutamide was used as an androgen receptor antagonist. (B) PSA release was stimulated by 50 pM R1881 exposure in LNCaP cells and measured 4 d after TQ or AT treatment. (C) PC3 cells co-transfected with an androgen receptor expression vector and an androgen-responsive GFP reporter stimulated with R1881. Cells treated with vehicle (left panel) and 25 μM TQ (right panel) showing R1881-stimulated, GFP expressing cells (arrows) (see MARS assay in Materials and Methods). (D) Quantification of GFP positive cells per well compared to control, vehicle treated samples in a dose-response of TQ from 5 to 50 μM. * *P*<0.05.

Studies to determine if TQ or AT modulated AR activity were initiated using an androgen-sensitive luciferase reporter system. For this study, androgen-sensitive reporter activity was stimulated using the synthetic androgen R1881 and was assessed after treatment with either 30 μM TQ or AT (Fig 3A). TQ treatment alone did not modulate reporter activity. In contrast, TQ was found to significantly inhibit R1881-induced reporter activation after 2 d in comparison to R1881-stimulated control cells. Surprisingly, 30 μM AT treatment increased androgen-sensitive reporter activity (Fig 3A). This data supports an inhibitory role for TQ on AR activity in contrast to AT, which did not exhibit antiandrogenic activity.

The release of prostate specific antigen (PSA; KLK3) from LNCaP cells is recognized as a sensitive indicator of androgenic response in LNCaP cells [17]. To further examine TQ’s effects on androgenic pathways, the androgen-stimulated release of PSA from LNCaP cells was determined. LNCaP cells treated with TQ showed a dose-dependent reduction in R1881-induced PSA release compared to untreated control cells (Fig 3B). In contrast, treatment with 10 to 40 μM AT did not affect androgen-induced PSA release from LNCaP cells (Fig 3B). Forced expression of the androgen receptor in PC3 human prostate cells to assess activation of an androgen-responsive green fluorescence reporter was used to measure the ability of TQ to inhibit androgen receptor activation. Control, vehicle-treated cells showed extensive androgen receptor activation after stimulation with the synthetic androgen R1881 (Fig 3C [left panel] and Fig 3D), whereas cells treated with 5, 10, 25, or 50 μM TQ showed a significant reduction in androgen receptor activation by R1881 (Fig 3C [right panel] and Fig 3D).

The decrease in PSA release may be due in part to down-regulation of *PSA* gene expression produced by TQ (Table 1). In addition to *PSA* mRNA levels, other androgen-responsive genes were measured after TQ treatment. As shown in Table 1, mRNA levels for the AR-responsive genes *kallikrein 2*, *prostein*, *prostatic acid phosphatase*, *NKX3*.*1* and *prostate specific membrane antigen* were reduced in LNCaP cells 4 d after treatment with TQ. In contrast to TQ, AT treatment did not significantly alter expression of androgen-sensitive mRNAs (Table 1).

**Table 1:** Down-regulation of Androgen-Responsive Gene Expression by TQ and AT.

Gene	Symbol^3^	Fold decrease in [mRNA]^1^^,^ ^2^	
		TQ	AT
*Transmembrane 4 L Six Family Member 1*	TM4SF1	22.2	1.1
*Prostate Specific Antigen (PSA)*^4^	KLK3	9.1	1.1
*Kallikrein-Related Peptidase 2*	KLK2	7.1	1.0
*FK506 Binding Protein 5*	FKBP5	2.9	1.3
*Prostein*	SLC45A3	2.6	1.2
*Acid Phosphatase*, *Prostate*	ACPP	2.4	1.2
*NK3 Homeobox 1*	NKX3-1	1.9	1.1
*Prostate-Specific Membrane Antigen*	FOLH1	1.5	1.1

^1^ Analysis by quantitative PCR (see Materials and Methods).

^2^ Compared to control, vehicle-treated LNCaP cells.

^3^ National Center for Biotechnology Information Gene Official Symbol (Name/Gene ID).

^4^ NCBI Official Full Name is kallikrein-related peptidase 3.

### Down-regulation of AR protein levels in androgen-responsive prostate cancer cells by TQ

**Fig 4:**
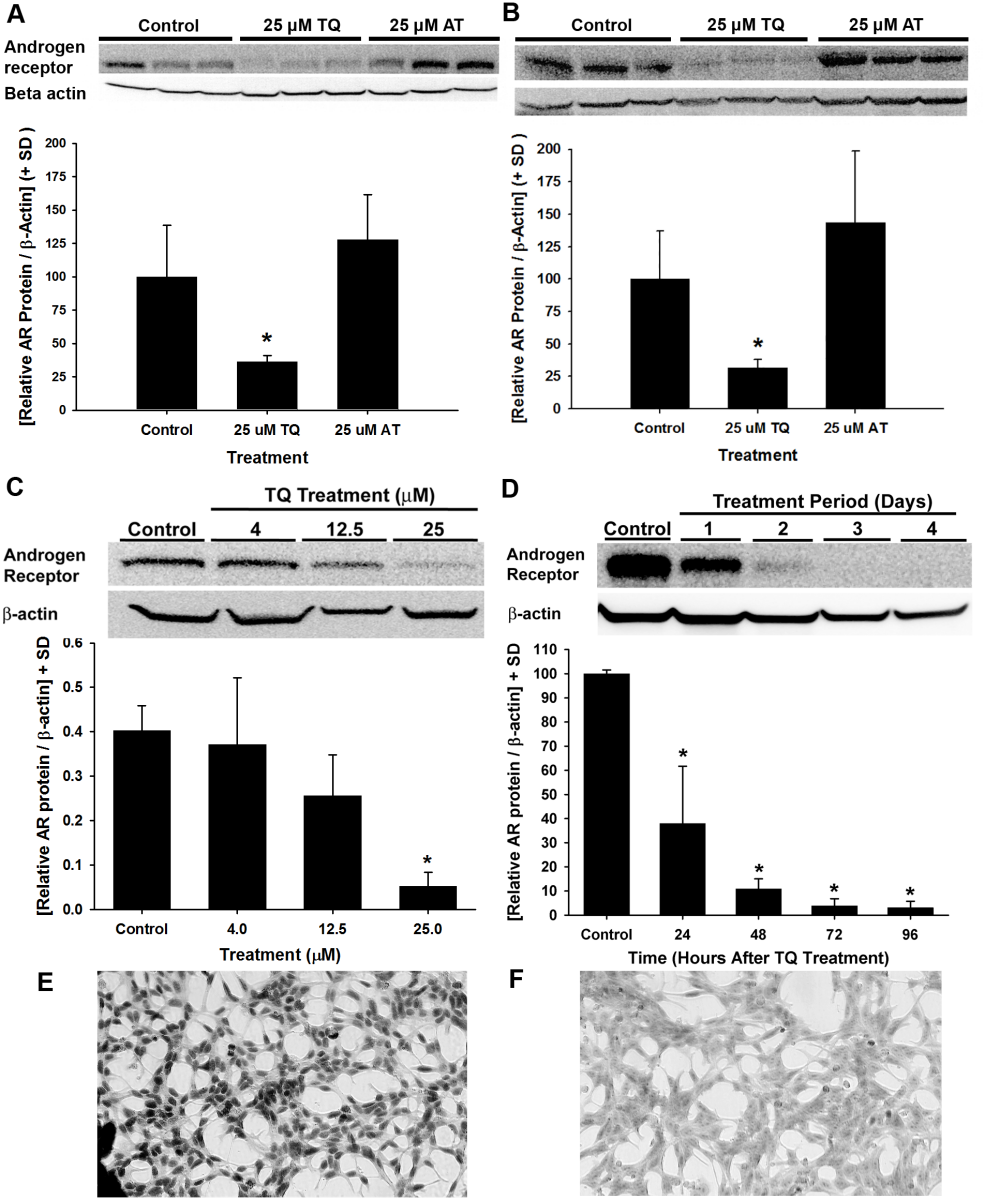
**A-F**. Cellular localization, dose-response, and time-course of TQ-induced AR protein down-regulation in AR-expressing prostate cancer cells. AR protein levels determined by immunoblot in LNCaP (A) and LAPC4 (B) cells treated with 25 μM TQ or 25 μM AT for 4 d. (C) TQ dose-dependent reduction in AR protein levels in LNCaP cells treated with 4, 12.5, or 25 μM TQ for 4 d. (D) Immunoblot of time-dependent changes in AR protein levels from LAPC4 cells treated with 25 μM TQ for 24, 48, 72, and 96 h. Quantified AR protein levels are presented below each immunoblot (* *P*<0.05). (E-F) LNCaP cells were treated with vehicle control (E) or 25 μM TQ (F) for 4 d and AR protein expression was detected by immunocytochemistry.

To determine the effects of AT and TQ on AR protein in androgen-responsive cells, immunoblot analysis for AR protein was performed. LNCaP and LAPC4 cells were treated with vehicle control, 25 μM TQ, or 25 μM AT for 4 d (Fig 4A and 4B). For both lines, cells treated with TQ showed a substantial reduction in AR protein in comparison to vehicle-treated controls, whereas AT showed no difference in AR detection in comparison to controls (Fig 4A and 4B). To better understand the dynamics of AR down-regulation by TQ in prostate cancer cells, the cellular AR localization, dose, and time required for TQ effects in LAPC4 and LNCaP cells were determined. A dose-response of 4, 12.5, or 25 μM TQ in LNCaP cells showed reduced AR protein levels with 25 μM after 4 d (Fig 4C). Similar to LNCaP cells, TQ significantly inhibited AR protein levels in LAPC4 cells treated with 25 μM TQ for 24, 48, 72, and 96 h (Fig 4D). Immunocytochemical staining of AR in LNCaP cells showed significant nuclear localization of the AR (Fig 4E), whereas cells treated with 25 μM TQ for 4 d showed intact cells with a significant reduction in the levels of AR protein staining (Fig 4F). Taken together, these results show that TQ, but not AT, has potent antiandrogenic effects on androgen-responsive prostate cancer cell lines.

### Determination of *AR* mRNA down-regulation by TQ and AT

**Fig 5:**
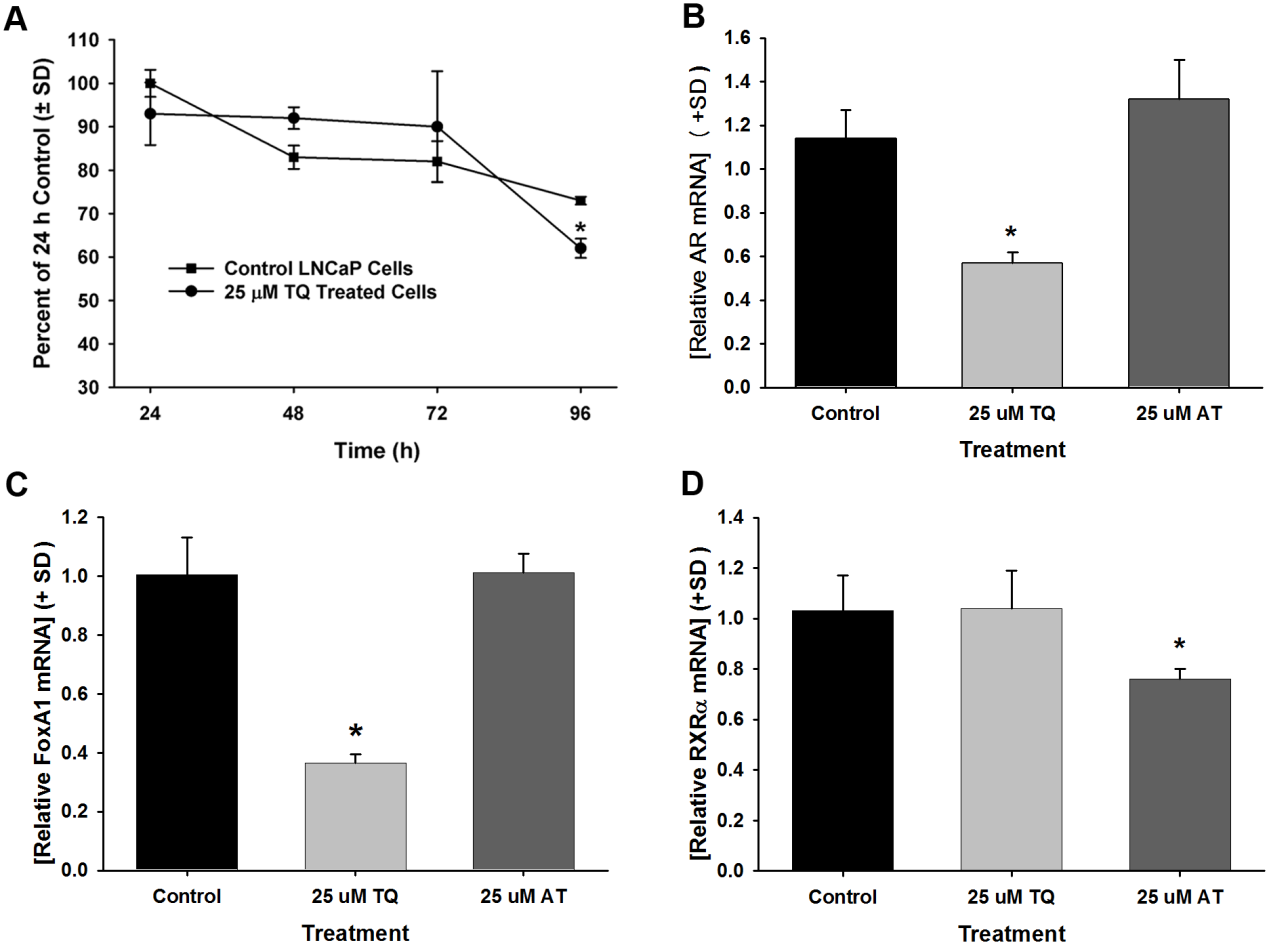
**A-D**. Expression of transcription factor mRNAs in TQ-treated LNCaP and LAPC4 cells. (A) Time course analysis of AR mRNA levels in TQ treated LNCaP cells compared to AR mRNA levels in control, vehicle treated LNCaP cells. (B) mRNA levels in LAPC4 cells treated for 4 d with 25 μM TQ or 25 μM AT compared to control, vehicle-treated cells. Levels of *FOXA*1 mRNA (C) and *RXR*α mRNA (D) in LNCaP cells treated for 4 d with 25 μM TQ or 25 μM AT compared to controls. * *P*<0.05.

We further evaluated TQ down-regulation of *AR* mRNA and demonstrate this action was distinct from AT. *AR* mRNA expression after TQ treatment for 24, 48, 72, and 96 h in LNCaP cells was determined showing a significant decrease in *AR* mRNA at 96 h (Fig 5A). Thus, the expression levels of *AR* mRNA in LAPC4 cells and additional mRNAs were measured after treatment with 25 μM TQ or AT for 4 d. *AR* mRNA levels were decreased 1.7-fold after treatment with 25 μM TQ in LAPC4 cells (Fig 5B). Note that reductions in AR protein levels (Fig 4D) were observed to occur prior to the reduction observed in *AR* mRNA. Furthermore, although a significant reduction of *AR* mRNA was seen at 4 d upon TQ treatment in both LNCaP and LAPC4 cells, by comparison to the relative down-regulation of *AR* mRNA at 4 d, AR protein was more significantly inhibited in matched samples (data not shown), suggesting that TQ down-regulation of AR protein expression may be, at least in part, independent of transcriptional inhibition of *AR* mRNA expression. TQ also significantly decreased *FOXA1* mRNA expression levels (Fig 5C). It is noteworthy that AT did not affect *AR* or *FOXA1* levels mRNA levels after 4 d (Fig 5A, 5B and 5C). However, mRNA down-regulation was not an overt action of TQ as *retinoid X receptor*, *alpha* (*RXRα*) mRNA levels were not changed by TQ in LNCaP cells (Fig 5D). Although AT treatment did not affect the mRNA levels of the androgen-responsive genes assessed, AT produced a 20% reduction in *RXRα* mRNA levels (Fig 5D), providing further support for differential effects of AT and TQ on prostate cancer cells.

### AR protein down-regulation by TQ was abrogated by antioxidants

**Fig 6:**
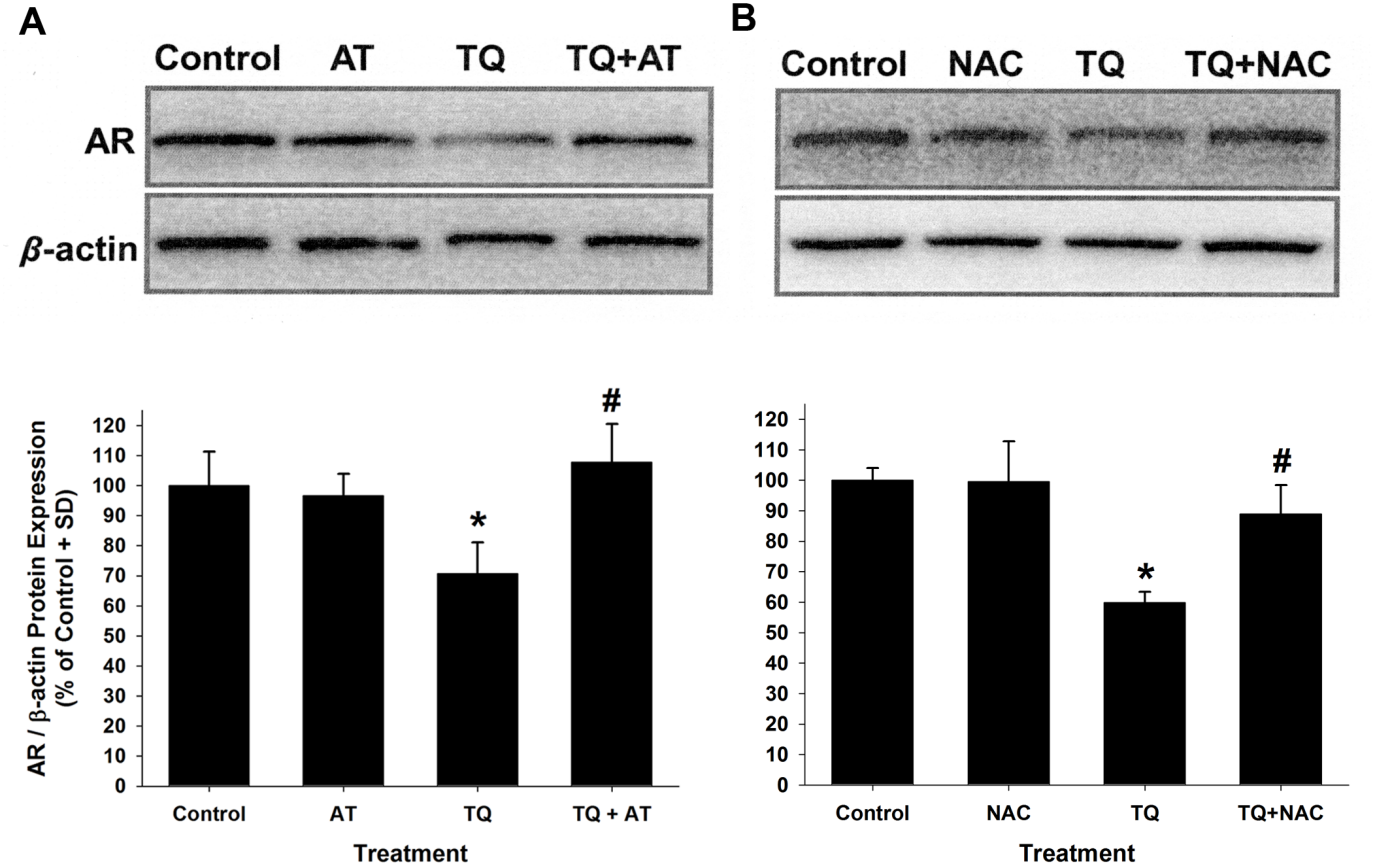
**A-B**. Inhibition of TQ-mediated AR protein down-regulation by the antioxidants AT and NAC in LNCaP cells. (A) Immunoblot analysis of AR protein expression in cells pretreated for 24 h with 25 μM AT and then treated with 25 μM TQ in the presence or absence of 25 μM AT for an additional 48 h. (B) Immunoblot analysis of AR protein levels in cells pretreated for 24 h with 5 mM NAC and then treated with 25 μM TQ in the presence or absence of 5 mM NAC for 48 h. (*P<0.05 compared to control; #*P*<0.05 compared to TQ-treated AR protein levels).

The antioxidants N-acetylcysteine (NAC) and AT were used to determine if a potential mechanism of TQ’s down-regulation of AR protein expression is affected by antioxidant treatment. LNCaP cells were pre-treated with 5mM NAC or 25μM AT for 24h and subsequently treated with 25μM TQ with or without AT (Fig 6A) and NAC (Fig 6B) for 48h. The TQ-mediated down-regulation of AR protein was found to be significantly attenuated by the presence of antioxidants, supporting that TQ may down-regulate AR protein through pathways that are sensitive to the action of these antioxidants.

## Discussion

The biological actions of AT have been investigated extensively. In contrast, much less is known about AT’s oxidation product, TQ. Here, the down-regulation of androgenic activity by TQ was investigated with the finding that this activity was dependent on TQ’s activity as a pro-oxidant. AT did not significantly affect either the growth of prostate cancer cells or pathways known to be critical in prostate cancer progression compared to TQ. This study begins to identify the novel anti-androgenic activity of TQ and demonstrates the differential activity of AT and TQ in human prostate cancer cells. TQ significantly inhibited androgen-responsive prostate cancer cell proliferation, AR activity, and AR protein expression. TQ was found to down-regulate AR protein expression in both a time- and dose-dependent manner through a mechanism involving alterations in cellular redox. We further demonstrated that TQ down-regulation of AR protein was attenuated by the antioxidants NAC and AT. The ability of these distinct antioxidants to abrogate the actions of TQ on AR protein in prostate cancer cells is the focus of future studies. Overall, a novel action for TQ was found in the down-regulation of androgenic activity in human prostate cancer cells.

In this study, neither the growth of prostate cancer cells nor the pathways known to be critical in prostate cancer progression that were compared were affected by AT, whereas TQ potently inhibited the growth of androgen-sensitive prostate cancer cells. The decrease in cell growth produced by TQ treatment may be AR-dependent as TQ treatment did not have a pronounced effect on the growth of the androgen-independent DU145 human prostate cancer cell line. Importantly, TQ, but not AT, was found to reduce both *AR* mRNA and AR protein levels in prostate cancer cells with a concomitant reduction in androgenic pathways. Several studies have shown that down-regulation of the AR results in decreased cell proliferation in androgen-sensitive prostate cancer cells. For example, decreased AR expression was achieved in LNCaP human prostate cancer cells using siRNA resulting in a decrease in LNCaP growth [18,19]. Thus, the decrease in cell growth produced by TQ in androgen-sensitive prostate cancer cell lines may be due at least in part to the action of TQ to down-regulate AR expression.

The AR is a tissue-specific, ligand-activated transcription factor that is known to regulate the expression of genes such as *transmembrane 4 L six family member 1*, *PSA*, *kallikrein 2*, *prostein*, *prostatic acid phosphatase*, *NKX3*.*1*, and *prostate specific membrane antigen* in prostate cells [20–24]. Because the AR plays a key role in maintenance of the expression of these genes, their expression would be reduced by interventions that down-regulate the AR. In fact, the expression of several of these genes was reduced after treatment of LNCaP cells with TQ. Additionally, expression from an androgen-sensitive reporter was inhibited by concurrent androgen and TQ treatment. In contrast, AT had minimal effects on the modulation of androgen-responsive genes or gene products. The reduced expression of AR-responsive genes induced by TQ treatment strongly supports that the AR is a major target of TQ in prostate cancer cells.

The AR is recognized as a major contributor to all stages of prostate cancer from carcinogenesis to castration-resistant disease [7,12,25,26]. To date, most interventions against prostate cancer reduce AR activation through inhibiting the production of androgenic ligands, such as testosterone or dihydrotestosterone. These strategies do not affect the AR itself. To modulate AR activity, it is necessary to identify interventions that target down-regulation of AR expression. Here, we show that down-regulation of AR protein and mRNA can be achieved using TQ with a pronounced impact on androgenic activity in prostate cancer cells. It is noteworthy that AT did not inhibit either AR expression or activity in prostate cancer cells. This is important as this is the form of AT that is expected to be physiologically active in contrast to ester conjugated forms, such as vitamin E succinate, that are purportedly converted to α-tocopherol by the action of esterases in cells and in the body.

Although AT did not exhibit anti-androgenic properties within our system, vitamin E (VE) analogs have been reported to affect AR protein expression in prostate cancer cells. For example, Zhang et al. [16] reported that the VE analog, VE succinate, reduces AR activity in androgen-sensitive human prostate cancer cells. Similar to TQ, VE succinate treatment was found to decrease both AR mRNA and protein levels in LNCaP cells [16]. Of importance, Zhang et al. [16] found that at least part of VE succinate’s action is due to a decrease in AR translation. Our laboratory has previously reported on the anti-androgenic activity of another VE analog, 2,2,5,7,8-pentamethyl-6-chromonol (PMCol) [14]. This antioxidant moiety of AT, PMCol, consists of the chromonal ring structure of AT but lacks the phytyl chain. We demonstrated that PMCol inhibited the proliferation of androgen-sensitive prostate cancer cells acting as a competitive inhibitor of AR ligand binding and that PMCol inhibits AR activation [14]. However, PMCol did not inhibit AR expression. Therefore, limitations of VE analogs may include the lack of metabolic conversion to AT and, hence TQ, or the lack of AR protein down-regulation.

Identifying the mechanism of TQ’s anti-androgenic activity and selective inhibition of AR protein expression may provide insight into novel AR regulatory mechanisms. Because TQ had pronounced inhibitory effect on markers of AR activity, the AR in androgen-sensitive prostate cancer cell lines was examined. Both AR protein and mRNA were found to be reduced by TQ treatment in both LAPC4 and LNCaP human prostate cancer cells. However there was significant reduction of AR protein expression that preceded the inhibition of AR mRNA expression, demonstrating that TQ’s actions on AR down-regulation may not be entirely due to the inhibition of AR mRNA expression. Interestingly, *FOXA1* mRNA was also reduced by TQ treatment. FOXA1 is recognized as a potent contributor to androgenic activity for prostate genes [27]. However, down-regulation of mRNA expression was not an overt action of TQ activity. For example, *RXRα* mRNA expression was not found to be affected by TQ treatment.

The actions of AT as a measure for alleviating prostate cancer are controversial. Due to discrepancies between epidemiologic studies regarding AT activity for prostate cancer, alternative explanations for the outcomes of these studies should be explored. For example, smoking was a major discrepancy between the participants of the Alpha-Tocopherol, Beta-Carotene Cancer Prevention (ATBC) trial [28], which enrolled heavy smokers, and the Selenium and Vitamin E Cancer Prevention Trial (SELECT) [29], which enrolled mostly non-smokers. This is intriguing when considering the prostate cancer preventive actions of supplemental AT when taken by men who smoke as seen between these two studies. For example, in the ATBC study, a 32% reduction in prostate cancer incidence and 41% reduction in mortality was observed among smokers taking supplemental AT compared to control groups [30]. In the Harvard Health Professionals study, no effect of supplemental AT alone was found on prostate cancer incidence; however it was reported that, “among current smokers and recent quitters, those who consumed at least 100 IU of supplemental AT per day had a relative risk of 0.44 for metastatic or fatal prostate cancer” [31]. Additional studies have found no effect of supplemental AT when taken alone, but did report a reduction in the development of prostate cancers among smokers taking VE supplements [28,32,33]. In contrast to these reports, a recent study has found that AT itself may have activity against the development of advanced prostate cancer [34]. These finding conflict with the results from the SELECT, which failed to find prostate cancer preventive actions of supplemental AT [29,35]. Thus, several studies to date suggest that AT itself may not be an effective intervention against prostate cancer. Supportive of these findings, the results from the current study did not find significant effects on prostate cancer cells by AT. However, we have found that TQ, the major oxidation product of AT, is highly effective at reducing both growth and androgenic activity in prostate cancer cell lines. It is intriguing to consider that TQ may be the active derivative of AT involved in prostate cancer prevention among heavy smokers taking supplemental VE, which in possessing a physiologic oxidative stress may effectively transform AT to TQ. The results from the current study strongly support further investigations to determine the efficacy of TQ as a modality for prostate cancer prevention.

The elevated detection of TQ levels upon AT supplementation and increased oxidative stress has been demonstrated in animal models. For instance, Wurzel, H et al. [36] conducted an *in vivo* study which exposed rats to chronic cigarette smoke and AT for 65 weeks. In the experimental group, they found high levels of TQ in the bronchoalveolar lavage fluid demonstrating that smoke-exposed animals generated a larger amount of oxidative products [36]. The detection of TQ in humans has been reported in a comparative study of adult pulmonary patients routinely receiving oxygen therapy [37]. The conversion of AT into TQ under oxidative insult could be a potential explanation for the discrepancy observed between the prevention trials previously mentioned.

Reports on the biological effects of TQ are limited. This may be due in part to TQ being regarded simply as the product of AT oxidation with limited inherent biological activity. However, TQ is chemically distinct from AT and, therefore, may have unique biological actions compared to AT. The distinct biological actions of TQ and AT are strongly supported by the results on selective AR down-regulation by TQ observed in the current study. A physiological action associated with TQ is anticoagulant activity [38]. This is not surprising in that the quinone and phytyl chain structure of TQ is reminiscent to that of vitamin K, a critical vitamin involved in blood clotting. In general, chemicals possessing quinone structures are found to be toxic. This is largely due to the presence of electrophilic carbon centers present in the quinone structure that may be acted upon by nucleophiles present in cellular constituents [3]. In the current study, TQ was not found to be highly cytotoxic. Interestingly, all electrophilic sites in TQ are blocked by methyl substitutions and thus TQ would be expected to be less reactive than chemicals with unblocked quinone structures. Additionally, TQ has been found to be a potent substrate for the biotransformation enzyme NQO1 [39]. The reduction of TQ to the hydroquinone by NQO1 was found to be so efficient it was suggested that TQ may be one of the primary substrates for NQO1’s biological activity [39]. Results from the current study and others strongly support that TQ has potent biological actions that are distinct from AT. Importantly, TQ’s down-regulation AR protein expression in human prostate cancer cells is mediated by alterations in cellular oxidative stress that can be abrogated by antioxidants.

## Materials and Methods

### Chemicals, cell culture, and treatment protocols

dl-α-tocopherylquinone was obtained from Research Organics (Cleveland, OH). Methyltrienolone (i.e., R1881) was obtained from Perkin Elmer/NEN Life Science Products (Boston, MA). Bicalutamide was from LKT Laboratories, St. Paul, MN. Vitamin E as dl-α-tocopherol and other chemicals used in these studies were from Sigma Chemical Co (St. Louis, MO).

The DU145, LNCaP, and PC3 cells used in these studies were acquired from American Type Culture Collection (Manassas, VA). Cells were maintained in Dulbecco’s modified Eagle’s medium (DMEM; Invitrogen, Carlsbad, CA) containing 5% heat-inactivated fetal calf serum (FCS; Sigma, St. Louis, MO) with streptomycin-penicillin antibiotics (designated DMEM/FCS) in a 5% CO_2_ incubator at 37°C. LAPC4 cells adapted to growth in DMEM-based medium (i.e., DMEM/FCS) and 5% FCS were acquired from Dr. George Wilding (University of Wisconsin Paul P. Carbone Comprehensive Cancer Center). For most experiments evaluating androgenic responses, cells were cultured in DMEM containing 4% charcoal-stripped FCS and 1% FCS (designated DMEM/CSS). Methods were developed to insure that TQ and VE could effectively be delivered to prostate cancer cells in culture. This was achieved using a carrier-based delivery method for TQ and VE dissolved first in ethanol which was added to a 7.5% bovine serum albumin (BSA) solution for a 20-fold concentrated stock. This solution was then added to standard growth medium at a 5% concentration (i.e., a final concentration of 0.4% BSA) to produce concentrations of AT in culture medium ranging from 10 to 40 μM.

### AT and TQ measurements in tissue culture medium

The addition of TQ and VE to medium was performed as described earlier. TQ and AT are composed of lipophilic hydrocarbon chains (Fig 1) that greatly limit their solubility in cell culture medium. Therefore, for these studies, methods were developed to effectively treat prostate cancer cells in culture with TQ and AT. Levels of TQ and AT in tissue culture medium were measured using an ESA high-performance liquid chromatography (HPLC) system (ESA, Inc., Chelmsford, MA) with a 250 mm AltechLiChrosorb RP-18 reverse-phase column, an ESA model 582 solvent delivery system, and an ESA CoulArray detector controlled by CoulArray Software for Windows. The mobile phase consisted of 5 mM sodium acetate and 5 mM acetic acid in HPLC grade methanol (Figure in S1 Fig).

### Cellular proliferation and cell foci assays

Cell growth analysis were performed using DU145, LNCaP, and LAPC4 cells plated in 96-well tissue culture plates. Relative cell numbers with and without TQ and VE treatment were determined using the CyQUANT NF Cell Proliferation Assay Kit (Invitrogen), according to kit instructions.

The number of foci formed in LNCaP cultures was determined as an additional measure of cell proliferation. LNCaP cells were treated with 10 μM TQ, 10 μM VE or BSA for 5 d. Cell foci formation were counted by light microscopy. Numbers of foci formed were determined within a 40× field of view.

### Cell viability analysis

Relative cell viability changes were determined using DU145, LNCaP, and LAPC4 cells plated in 12-well tissue culture plates. Cell count and viability was determined by trypan blue dye (0.4%; Sigma, St. Louis, MO) exclusion using a hemocytometer and light microscopy. Additionally, cell viability was determined in vehicle and 25 μM TQ-treated DU145 and LNCaP cells using 1 μg/mL propidium iodide (Sigma) exclusion measured using a BD Accuri C6 Flow Cytometer with BD Accuri C6 Software (BD Biosciences, Ann Arbor, MI).

### Immunocytochemical AR analysis

LNCaP cells were treated with 25 μM TQ, 25 μM AT or vehicle control for 4 d. Cells were grown and treated on coverslips. Slides were then fixed with 3.7% formaldehyde for 1 min, methanol for 1 min, and then blocked with 2.5% goat serum for 30 min. Primary antibody anti-AR (441) was detected using the secondary horseradish peroxidase-conjugated goat anti-mouse IgG. Slides were then incubated with 3′,3′-diaminobenzidine (Sigma) and counterstained using hematoxylin.

### AR protein immunoblot analysis

LNCaP and LAPC4 cells were plated at a density of 1×10^6^ cells per 100 mm cell culture plate in 10 ml of DMEM/CSS and maintained in incubators at 37°C in 5% CO_2_. For dose-response studies, LNCaP cells were cultured in 6-well plates (BD Biosciences, San Jose, CA) in DMEM containing 5% FBS. After a 4 d treatment with vehicle, AT, or TQ, cells were washed in cold 1× PBS and lysed in a buffer containing 1.0% Igepal CA-630, 0.5% sodium deoxycholate, 0.1% sodium dodecyl sulfate, 0.1 mg/ml phenylmethylsulfonyl fluoride, 1 mM sodium orthovanadate, and 10 μg/ml aprotinin in 1× PBS. Cell extracts were stored at -80°C until analysis. Sample protein levels were determined using the BCA Protein Assay kit (Pierce Biotechnology, Rockford, IL), according to kit instructions. Total protein (25 to 30 μg) from cell extracts were electrophoresed on 12.5% SDS-polyacrylamide gels (BioRad, Hercules, CA) and transferred to Immobilon-P membranes (Millipore Corp., Bedford, MA) using a GENIE wet transfer system (Idea Scientific, Minneapolis, MN). Membranes were blocked in Tris-buffered saline containing 5% nonfat dry milk at 4°C and then incubated with mouse anti-AR (441) monoclonal antibody (Santa Cruz Biotechnology, Santa Cruz, CA) or mouse anti-β-actin antibody (A5441; Sigma). After washing, membranes were incubated with a secondary horseradish peroxidase-conjugated goat anti-mouse IgG (Biomeda, Foster City, CA) and analyzed using Western Lightening Chemiluminescence Reagent Plus (Boston, MA) on a Kodak Image Station 4000MM (Rochester, NY). Band intensities were determined using Kodak Molecular Imaging Software for the androgen receptor band at R_f_ 100 to 120 kD.

### Messenger RNA expression analysis

Total RNA was extracted from cells using TRIzol Reagent (Invitrogen) and cDNA was prepared from total RNA using the High Capacity cDNA Reverse Transcription Kit (Applied Biosystems, Foster City, CA). Quantitative PCR (qPCR) was performed for mRNA levels using an Applied Biosystems 7900HT Fast Real-Time PCR System (Carlsbad, CA) and QuantiTect Primers Assays (Qiagen Inc., Valencia, CA) for *AR*, *NQO1* and *GAPDH* mRNA. Additional forward and reverse primers used for qPCR are listed in Table in S1 Table.

### Prostate specific antigen analysis

LNCaP cells were cultured in 96-well plates at 5x10^3^ cells per well in DMEM/CSS 1 d before treatment. After a 4 d treatment with 50 pM R1881 and TQ or VE, media levels of PSA released from LNCaP cells were measured using a PSA Enzyme Immunoassay Test Kit (BioCheck, Inc., Foster City, CA) according to the kit’s instructions. PSA levels were normalized to cell number, which were determined using the CyQUANT NF Cell Proliferation Assay Kit (Invitrogen) as described above.

### Reporter activation assay

LNCaP cells were cultured in 12- or 24-well plates (Invitrogen) in DMEM/CSS 2 to 3 d before transfection. Androgen-induced transcriptional activation was determined using a reporter construct with an androgen-sensitive MMTV-LTR that regulates the expression of luciferase [40]. Cells were transfected using the calcium phosphate precipitation method with the MMTV/luciferase plasmid [40]. Twenty-four h after transfection, cells were treated with R1881 with or without test reagents at the specified concentrations. Cell extracts were acquired after treatment in 100 μL of Cell Culture Lysis Reagent (Promega, Madison, WI). Luciferase activity was measured using the Luciferase Assay Substrate (Promega) and relative luciferase units determined on a TD-20/20 Luminometer (Turner Designs, Sunnyvale, CA).

### Multifunctional Androgen Receptor Screening assay

The Multifunctional Androgen Receptor Screening (MARS) assay, as described previously [41], was used to determine forced AR activity in PC3 human prostate cancer cells. PC3 cells were cultured in 96-well plates and, after 48 h, transfected with pDsRedhAR and pMMTVdsEGFP at a ratio of 1:20 using FuGENE 6 according to the manufacturers’ instructions (Promega, Madison, WI). Eight h after transfection, cells were treated with vehicle or 5, 10, 25, or 50 μM TQ for 48 h and then AR activity was stimulated by the addition of 5 nM R1881 (Perkin Elmer, Waltham, MA). Twenty-four h after R1881 treatment, the number of GFP positive cells per a well were quantified using an Olympus IX70 Inverted Microscope with fluorescent excitation at 488 nm.

### Statistical analyses

Significant differences in values between groups were assessed using an unpaired *t*-test with SigmaPlot 13 software (Systat Software, Inc., San Jose, CA). *P* values less than 0.05 were used to signify statistical significance. Independent studies were performed as specified with a minimum of 3 samples (i.e., n ≥3) per an experiment and each experiment was replicated.

## Supporting Information

S1 Fig AT and TQ measurements in tissue culture medium. The addition of AT and TQ to medium was performed as described in *Materials and Methods*. (A) AT retention time and signal response plot. (B) TQ retention time and signal response plot. (C) Plot of AT concentration versus peak area. Levels of AT and TQ in tissue culture medium were measured using an ESA high-performance liquid chromatography (HPLC) system (ESA, Inc., Chelmsford, MA) with a 250 mm AltechLiChrosorb RP-18 reverse-phase column, an ESA model 582 solvent delivery system, and an ESA CoulArray detector controlled by CoulArray Software for Windows. The mobile phase consisted of 5 mM sodium acetate and 5 mM acetic acid in HPLC grade methanol.
(PDF)

S1 Table Quantitative PCR Primer Sequences (PDF)
