# Supplementary figures and images for: Antioxidants Abrogate Alpha-Tocopherylquinone-Mediated Down-Regulation of the Androgen Receptor in Androgen-Responsive Prostate Cancer Cells
Source: PLoS One. 2016 Mar 17;11(3):e0151525. doi: 10.1371/journal.pone.0151525 (PMC4795544; doi:10.1371/journal.pone.0151525)

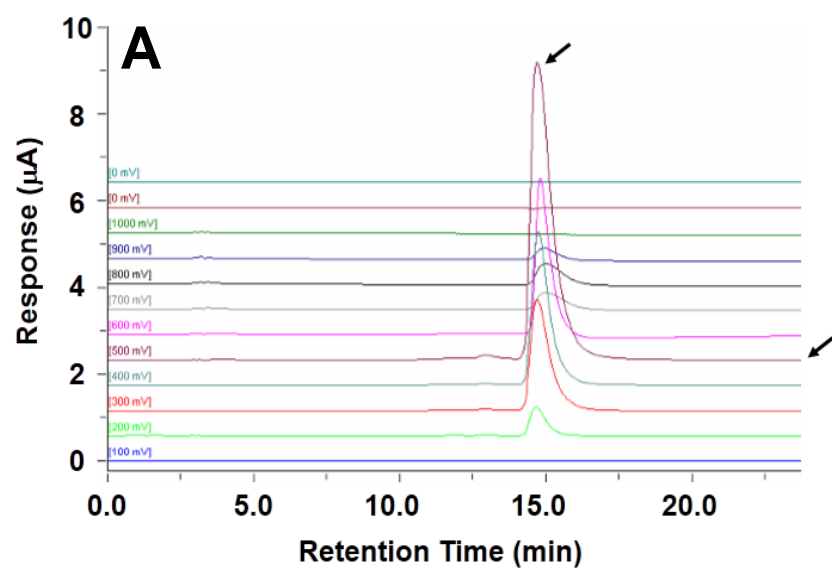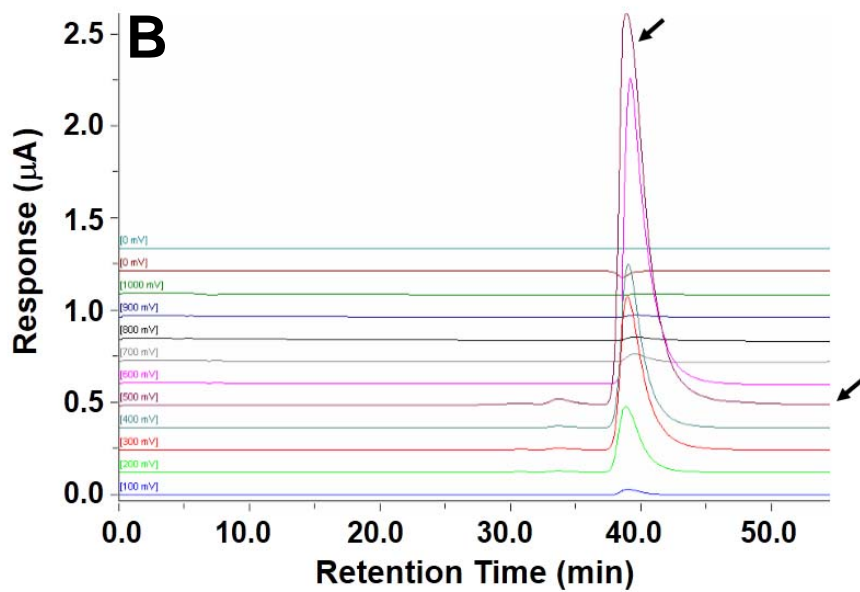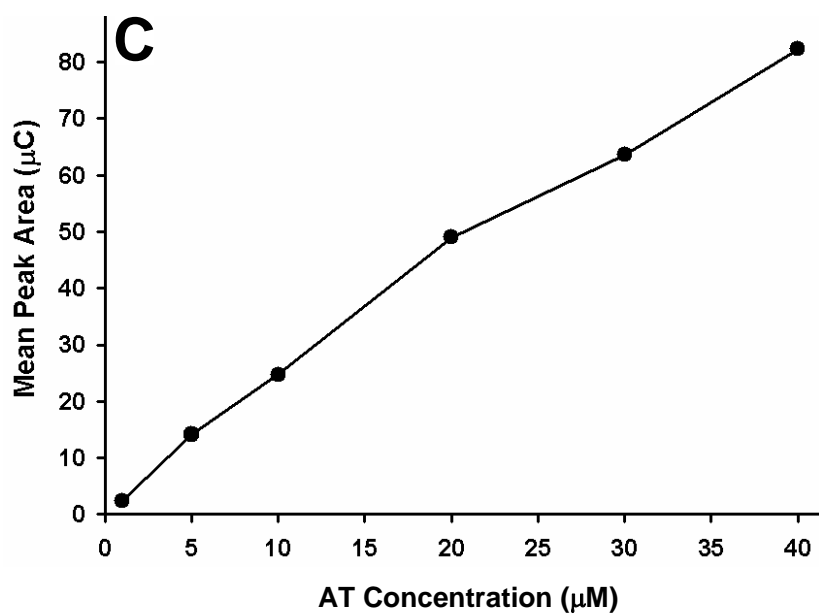

Supplement: S1 Fig — The addition of AT and TQ to medium was performed as described in Materials and Methods. (A) AT retention time and signal response plot. (B) TQ retention time and signal response plot. (C) Plot of AT concentration versus peak area. Levels of AT and TQ in tissue culture medium were measured using an ESA high-performance liquid chromatography (HPLC) system (ESA, Inc., Chelmsford, MA) with a 250 mm AltechLiChrosorb RP-18 reverse-phase column, an ESA model 582 solvent delivery system, and an ESA CoulArray detector controlled by CoulArray Software for Windows. The mobile phase consisted of 5 mM sodium acetate and 5 mM acetic acid in HPLC grade methanol. (PDF) [file pone.0151525.s001.pdf]
