# Supplementary material for: Antioxidants Abrogate Alpha-Tocopherylquinone-Mediated Down-Regulation of the Androgen Receptor in Androgen-Responsive Prostate Cancer Cells
Source: PLoS One. 2016 Mar 17;11(3):e0151525. doi: 10.1371/journal.pone.0151525 (PMC4795544; doi:10.1371/journal.pone.0151525)
Supplement: S1 Table — (PDF) [file pone.0151525.s002.pdf]

Supplemental Material

Table S1: Quantitative PCR primer sequences

Gene (Abbreviation)	Primer Direction	Primer Sequence (from 5′ to 3′)
Androgen Receptor (AR)	Forward	TCCTGGACTCCGTGCAGCCTATT
Androgen Receptor	Reverse	CCGGAAAGTCCACGCTCACCAT
FK506 Binding Protein 5 (FKBP5)	Forward	GAACCTGGCCATGTGCTACCTGA
FKBP5	Reverse	GCTGGGCTTCACCCCTCCTAT
Forkhead Box A1(FOXA1)	Forward	GCATGAAACCAGCGACTGGAACA
Forkhead Box A1	Reverse	GCTGACCGGGACGGAGGAGTA
Kallikrein 2 (KLK2)	Forward	CTGGGCTCTGGACAGGTGGTAAA
Kallikrein 2	Reverse	TACAGACAAGTGGACCCCCAGAAT
NKX3.1 (NKX3-1)	Forward	GGCCGAGACGCTGGCAGAGA
NKX3.1	Reverse	GGGCGCCTGAAGTGTTTTCAGAGT
Prostate Specific Antigen (PSA; KLK3)	Forward	CGCTGGACAGGGGGCAAAA
Prostate Specific Antigen	Reverse	ACAAGTGGGCCCCCAGAATCA
Prostate Specific Membrane Antigen (PSMA; FOLH1)	Forward	TCAGTGAGAGACTCCAGGACTTTGACA
PSMA	Reverse	GTTGTGGCTGCTTGGAGCATAGAT
Prostatic Acid Phosphatase (ACPP)	Forward	CTTCTTGCCACTTGACGGAATTGT
Prostatic Acid Phosphatase	Reverse	GTGCTGCGTCTCATTCCGGTAGTA
Prostein (SLC45A3)	Forward	CCTCCCTCTACCACCGGGAGAA
Prostein	Reverse	CCCTCGGTATTTGGGCAGGAA
Retinoid X Receptor, alpha (RXRα)	Forward	GTGGAGGCGCTGAGGGAGAA
Retinoid X Receptor, alpha	Reverse	GGCAGGCGGAGCAAGAGCTTA
TM4SF1	Forward	CCCTCGGCCAGTGGAACTACA
TM4SF1	Reverse	GCACTCGGACCATGTGGAGGTAT
